# Anti-cancer immune responses to DNA damage response inhibitors: Molecular mechanisms and progress toward clinical translation

**DOI:** 10.3389/fonc.2022.998388

**Published:** 2022-10-06

**Authors:** Lindsey Carlsen, Wafik S. El-Deiry

**Affiliations:** ^1^ Laboratory of Translational Oncology and Experimental Cancer Therapeutics, The Warren Alpert Medical School, Brown University, Providence, RI, United States; ^2^ The Joint Program in Cancer Biology, Brown University and the Lifespan Health System, Providence, RI, United States; ^3^ Department of Pathology and Laboratory Medicine, The Warren Alpert Medical School, Brown University, Providence, RI, United States; ^4^ Pathobiology Graduate Program, The Warren Alpert Medical School, Brown University, Providence, RI, United States; ^5^ Cancer Center, The Warren Alpert Medical School, Brown University, Providence, RI, United States; ^6^ Department of Medicine, Hematology-Oncology Division, Rhode Island Hospital, Brown University, Providence, RI, United States

**Keywords:** DNA damage response (DDR), immunotherapy, cGAS/STING, DNA-PK, WEE1, CHK1/2, ATR, ATM

## Abstract

DNA damage response inhibitors are widely used anti-cancer agents that have potent activity against tumor cells with deficiencies in various DNA damage response proteins such as BRCA1/2. Inhibition of other proteins in this pathway including PARP, DNA-PK, WEE1, CHK1/2, ATR, or ATM can sensitize cancer cells to radiotherapy and chemotherapy, and such combinations are currently being tested in clinical trials for treatment of many malignancies including breast, ovarian, rectal, and lung cancer. Unrepaired DNA damage induced by DNA damage response inhibitors alone or in combination with radio- or chemotherapy has a direct cytotoxic effect on cancer cells and can also engage anti-cancer innate and adaptive immune responses. DNA damage-induced immune stimulation occurs by a variety of mechanisms including by the cGAS/STING pathway, STAT1 and downstream TRAIL pathway activation, and direct immune cell activation. Whether or not the relative contribution of these mechanisms varies after treatment with different DNA damage response inhibitors or across cancers with different genetic aberrations in DNA damage response enzymes is not well-characterized, limiting the design of optimal combinations with radio- and chemotherapy. Here, we review how the inhibition of key DNA damage response enzymes including PARP, DNA-PK, WEE1, CHK1/2, ATR, and ATM induces innate and adaptive immune responses alone or in combination with radiotherapy, chemotherapy, and/or immunotherapy. We also discuss current progress in the clinical translation of immunostimulatory DNA-damaging treatment regimens and necessary future directions to optimize the immune-sensitizing potential of DNA damage response inhibitors.

## Graphical Abstract

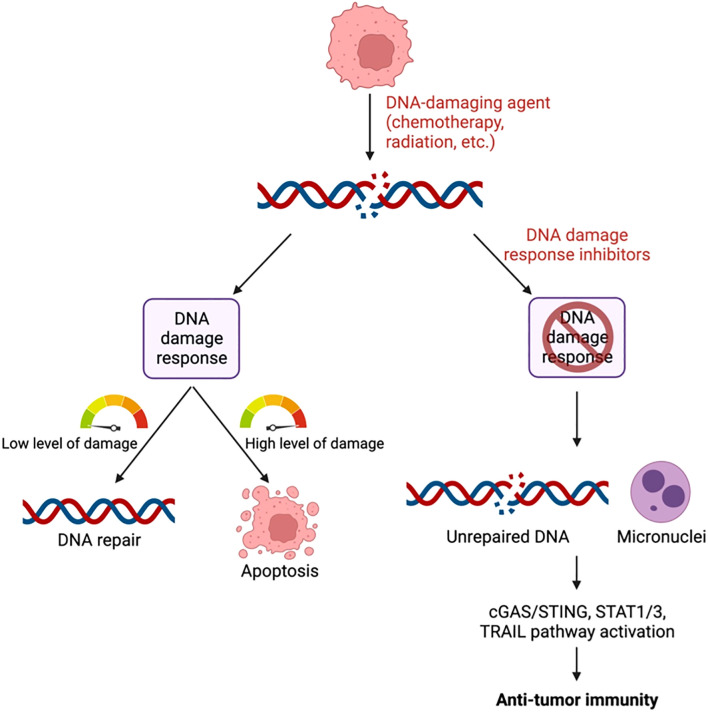

## Introduction

**Figure 1:**
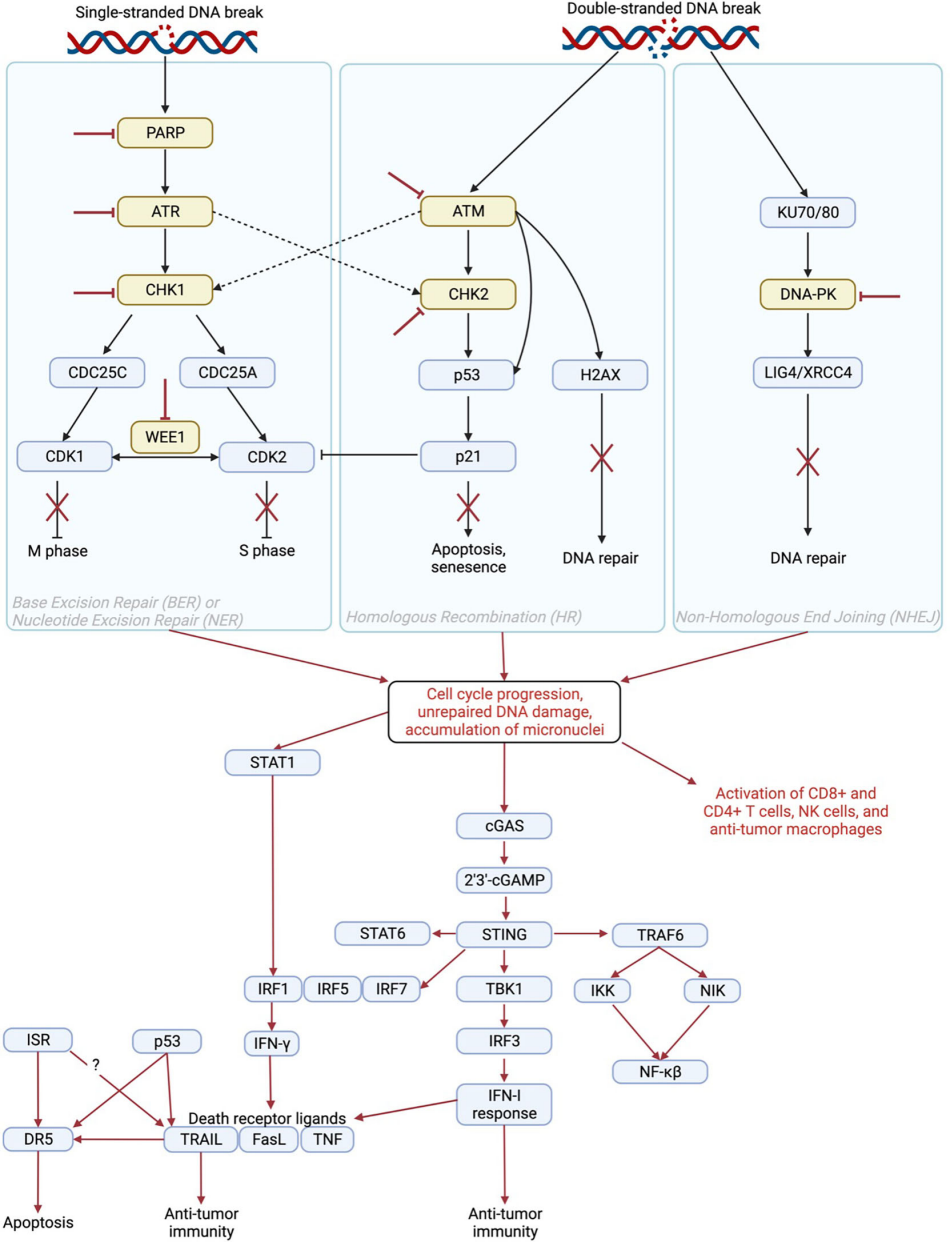
Mechanism of immune activation by inhibition of DNA damage repair proteins. PARP, ATR, CHK1/2, WEE1, ATM, and DNA-PK play roles in DNA repair pathways including base excision repair (BER), nucleotide excision repair (NER), homologous recombination (HR), and non-homologous end joining (NHEJ) to induce cell cycle arrest, apoptosis, senescence, and/or DNA repair. Inhibition of DNA damage repair proteins (red inhibitor lines) results in cell cycle progression, unrepaired DNA damage, and accumulation of cytosolic micronuclei that contain fragments of DNA (1). This results in activation of the STAT1 (2, 3), cGAS/STING (4), and TRAIL pathways as well as direct activation of immune cells (5, 6) to induce an anti-tumor immune response. The STAT1 pathway induces IFN-γ, which can increase levels of death receptor ligands including TRAIL (7), FasL (8), and TNF (9). The cGAS/STING pathway induces an IFN-I response, which also contributes to increased levels of death receptor ligands (10–13) as well as contributes directly to anti-tumor immunity (14). Created in BioRender.com.

The DNA damage response (DDR) involves several pathways including base excision repair (BER) and nucleotide excision repair (NER) to repair single-stranded DNA breaks as well as homologous recombination (HR) and non-homologous end joining (NHEJ) to repair double-stranded DNA breaks. Activation of these pathways results in cell cycle arrest, DNA repair, senescence, and/or apoptosis depending on the extent of DNA damage (**Figure 1**) (15). Inhibition of DDR proteins including poly-ADP ribose polymerase (PARP), DNA-dependent protein kinase (DNA-PK), WEE1, checkpoint kinase 1/2 (CHK1/2), ataxia telangiectasia and Rad3 related (ATR), or ataxia telangiectasia mutated (ATM) serine/threonine kinase results in cell cycle progression and accumulation of unrepaired DNA (16). This accumulation eventually leads to cell death and/or DNA leakage into the cytosol in the form of micronuclei (17). DDR inhibitor (DDRi) therapy is used to treat cancer patients with tumors that harbor alterations in DDR proteins such as BRCA1/2. In these tumors, inhibition of additional DDR proteins renders the cell incapable of any type of DNA repair, resulting in cell death (18). This mechanism is known as synthetic lethality, a situation in which inhibition or mutation of two proteins separately is viable, but mutation or inactivation of both is lethal to the cell (19). Even in the absence of DDRi agents, cancer cells with defects in DNA repair pathways tend be more sensitive to anti-cancer therapies (20) including chemotherapy as compared to cells without genetic alterations in these pathways (21, 22). In addition, cells with DNA damage repair defects tend to be sensitive to immunotherapy as a result of enhanced neoantigen generation, upregulation of programmed death ligand 1 (PD-L1), and induction of the cyclic GMP–AMP synthase (cGAS)/stimulator of interferon genes (STING) pathway (23–26).

DDRi therapy may be used as a single agent or in combination with DNA-damaging agents such as chemotherapy and radiation therapy (RT) (1) (**Table 1**). Certain PARP inhibitors (PARPi) are FDA-approved to treat breast, prostate, and gynecologic cancers including ovarian cancer (21–23), and there are numerous clinical trials underway to extend their use to other malignancies (**Table 1**). The WEE1 inhibitor ZN-c3 has been granted fast track designation by the FDA for treatment of patients with uterine serous carcinoma (27, 28) and is included in nine other clinical trials testing its efficacy in various other types of cancer. Additional clinical trials are ongoing to investigate other DDRi therapies including inhibitors of DNA-PK, and CHK1/2, ATR, and ATM.

**Table 1:** Ongoing, completed, and recruiting clinical trials testing the combination of DNA damage inhibitors with immunotherapy in various cancer types.

DDR target	Interventions	Cancer type	Phase	Trial #
**PARP**	Niraparib + Dostarlimab + RT	TNBC	II	NCT04837209
	Pembrolizumab + Olaparib	Cervical cancer, cervical carcinoma	II	NCT04483544
	Olaparib +/- Pembrolizumab	Metastatic pancreatic adenocarcinoma, stage IV pancreatic cancer AJCC v8	II	NCT04548752
	Olaparib + Durvalumab +/- Carboplatin, Etoposide, and/or RT	Extensive stage lung small cell carcinoma, stage IV, IVA, IVB lung cancer AJCC v8	I/II	NCT04728230
	Olaparib +/- Tremelimumab	Recurrent ovarian, fallopian tube or peritoneal cancer	II	NCT04034927
	Pembrolizumab + Olaparib	Breast cancer	II	NCT03025035
	Durvalumab + Olaparib + RT	Locally advanced, unresectable pancreatic adenocarcinoma, stage II & III pancreatic cancer AJCC v8	I	NCT05411094
	Durvalumab + Olaparib	Metastatic TNBC	I	NCT03544125
	Atezolizumab +/- Niraparib & Temozolomide	Advanced solid tumors	I/II	NCT03830918
	Olaparib +/- Atezolizumab	BRCA mutant non-HER2-positive breast cancer	II	NCT02849496
	Olaparib+ Pembrolizumab + Paclitaxel	Recurrent/advanced gastric and gastro-esophageal junction cancer with HRR mutation and MSS	I/II	NCT04592211
	Durvalumab + Olaparib + Copanlisib HCl	Advanced solid tumors with selected mutations	I	NCT03842228
	Durvalumab + Olaparib	Prostate cancer with high neoantigen load	II	NCT04336943
	Niraparib + Dostarlimab	BRCA-mutated unresectable or metastatic breast, pancreas, ovary, fallopian tube, or primary peritoneal cancer	I	NCT04673448
	Cabazitaxel + Carboplatin + Cetrelimab + Niraparib	Metastatic prostate cancer	II	NCT04592237
	Atezolizumab + Talazoparib	SLFN11 + small cell lung cancer	II	NCT04334941
	Cediranib Maleate + Durvalumab + Olaparib	Ovarian, primary peritoneal, or fallopian tube cancer after Pt therapy	II	NCT04739800
	Niraparib + Dostarlimab	HPV-negative head and neck squamous cell carcinoma	II	NCT04681469
	Olaparib + Tremelimumab	BRCA-deficient ovarian cancer	I/II	NCT02571725
	Dostarlimab + Niraparib	BRCA1/2 and PALB2-mutated metastatic pancreatic cancer	II	NCT04493060
	Rucaparib + Nivolumab	Solid tumors	II	NCT03824704
	NK cells + Talazoparib	Acute myeloid leukemia	I/II	NCT05319249
	Olaparib + Pembrolizumab	Advanced melanoma with homologous recombination mutation	II	NCT04633902
	Paclitaxel + Olaparib + Pembrolizumab	Advanced gastric adenocarcinoma	II	NCT04209686
	Busulfan + Gemcitabine + Melphalan + Olaparib + Rituximab + Vorinostat	Relapsed or refractory lymphomas undergoing stem cell transplant		NCT03259503
**DNA-PK**	M3814 + Avelumab +/- RT	Solid tumors	I	NCT03724890
	Avelumab + RT +/- Peposertib	Advanced/metastatic solid tumors and hepatobiliary malignancies	I/II	NCT04068194
	Radium 223 dichloride alone, + Peposertib, or + Peposertib and Avelumab	Advanced prostate cancer not responsive to hormonal therapy	I/II	NCT04071236
**WEE1**	Adavosertib + Durvalumab	Advanced solid tumors	I	NCT02617277
	ZN-c3 + Pembrolizumab (27)	Solid tumors		NCT04158336
**ATR**	Elimusertib + Pembrolizumab + RT	Recurrent head and neck cancer	I	NCT04576091
	M1774 + immune checkpoint inhibitor	Metastatic or locally advanced unresectable solid tumors	I	NCT05396833
	Elimusertib + Pembrolizumab	Advanced solid tumors	I	NCT04095273
**ATM**	None			
**CHK1/2**	None			

Search performed on 7/10/2022 using keywords “immunotherapy” and “PARP inhibitor, DNA-PK inhibitor, WEE1 inhibitor, ATR inhibitor, ATM inhibitor, or CHK1/2 inhibitor”.

In addition to inducing cancer cell death by synthetic lethality, it is now well-recognized that DDRi therapy induces innate and adaptive immune responses (29, 30). DDRi-induced immune stimulation primarily occurs *via* the cGAS/STING pathway (29), but also occurs through signal transducer and activator of transcription 1 (STAT1) pathway activation (2) and direct activation of immune cells including T cells, NK cells, and anti-tumor macrophages (5, 6). As a result of extensive preclinical evidence supporting DDRi-induced immune responses, several clinical trials have been initiated to test the combination of DDRi with immunotherapy, primarily immune checkpoint inhibition (ICI) (**Table 1**). In this Review, we will discuss the mechanisms of different DDR proteins, their interactions with the immune system, and clinical translation of DDRi + immunotherapy. We also discuss necessary future directions for optimal clinical translation including clarification of variation across different DDRi therapies and across cancer types, as well as the need for a stronger focus on combining DDRi + immunotherapy strategies with DNA-damaging agents such as chemotherapy and RT.

## cGAS/STING pathway

The cGAS/STING pathway is heavily implicated in the immunomodulatory effects of DNA damaging drugs and DDRi therapies. The first step of the pathway involves cGAS interaction with double-stranded DNA in the cytosol (31). These segments of DNA are often referred to as micronuclei (32). Then, cGAMP acts as a second messenger to activate STING, which activates TBK1 to recruit and activate IRF3. IRF3 then translocates to the nucleus to induce transcription of immune-stimulated genes (ISG) and type 1 interferons (IFNs). STING also activates IKK and NIK to mediate the induction of canonical and non-canonical NF-κB-driven inflammatory genes (31).

cGAS/STING-mediated IFN signaling enhances the infiltration of anti-tumor T cells and NK cells into the tumor. Though further study is needed to confirm this mechanism, it is also thought that cytosolic DNA from tumor cells can be transferred to the cytosol of immune cells to induce cGAS/STING signaling and enhance antigen presentation and cross-priming in DCs and T cells, respectively (31). Lastly, c-GAS-STING also promotes the senescence-associated secretory phenotype (SASP), which is characterized by cancer cell secretion of pro-inflammatory cytokines, chemokines, proteases, and growth factors that induce senescence and tumor control (31). However, it is important to mention that SASP can also induce an immunosuppressive TME, promoting cancer progression (33).

It is important to note that the effects of cytosolic DNA on cancer progression are likely dependent on cancer stage. In early stages, cytosolic DNA likely leads to immune surveillance through mechanisms such as the cGAS/STING pathway. In late stages, cancer cells are more likely to have lost functional checkpoints of cell cycle and immune regulation, and therefore cytosolic DNA can induce chronic inflammatory signaling that may be associated with survival and metastasis. Thus, the tumor microenvironment should be carefully monitored during the therapeutic induction of cytosolic DNA accumulation and cGAS/STING pathway activation using DDRi therapy (31).

STING agonists are being investigated to treat many types of cancer either as a single agent or combined with ICI or chemotherapy (34). STING-based therapeutics have yet to be combined with DDRi therapy, though there is rationale for combination to enhance DDRi-induced immune activation (34, 35). STING agonists can activate the cGAS/STING pathway in the absence of cytosolic DNA and therefore circumvent the need for DNA damage to induce the type 1 IFN response (35). Some limitations of targeting cGAS/STING in cancer exist, including evidence of cGAS/STING silencing or loss-of-function mutations in certain tumors (35, 36) and cGAS/STING-driven IL-6-dependent survival of chromosomally instable cancers (37). In these cases, administration of cGAS/STING agonists may have limited to no efficacy or may be pro-tumorigenic. Careful consideration of STING agonist combination therapies and evaluation of patients who may not benefit or be harmed by these therapies is needed prior to clinical translation of cGAS/STING + DDRi combination therapies.

## TRAIL pathway

The tumor necrosis factor (TNF)-related apoptosis-inducing ligand (TRAIL) is primarily expressed on the surface of immune cells including NK cells, T cells, NK tumor (NKT) cells, DCs, and macrophages (38). It can also be expressed in soluble form after proteolytic cleavage from the cell surface (39). Both membrane-bound and soluble TRAIL bind to death receptor 5 (DR5) and death receptor 4 (DR4) on cancer cells to induce apoptosis (39). TRAIL is induced by the IFN-γ/STAT1 and cGAS/STING pathways (7, 40–42) that are activated after DDRi therapy. The TRAIL pathway is anti-tumorigenic, as evidenced by the increased susceptibility of TRAIL receptor-deficient mice to chronic inflammation and tumorigenesis (43, 44).

In addition to its role in apoptosis, TRAIL also plays an important role in the anti-cancer immune response. For example, some immune cells kill cancer cells in a TRAIL-dependent manner (38, 45) and targeted delivery of TRAIL to cell surface antigens on T cells may enhance their cytotoxic activity (46). TRAIL-TRAIL receptor interaction on MDSCs can limit their lifespan, supporting an anti-tumor immune microenvironment (47, 48). Due to its ability to induce both apoptosis and anti-tumor immune responses, activation of the TRAIL pathway is a promising clinical strategy (49). Various therapeutic approaches have been considered including TRAIL receptor agonists, DR4/5 agonistic monoclonal antibodies, and different formulations such as PEGylated TRAIL (49). Other exciting new directions are being pursued preclinically such as engineering tumor-homing, TRAIL-expressing mesenchymal stem cells (50, 51). TRAIL-based therapies have been studied extensively in the clinic and some have shown early signs of efficacy in non-Hodgkin’s lymphoma (52) and non-small cell lung cancer (53, 54). Limitations include short half-life, limited induction of receptor clustering, binding to decoy receptors such as DcR1, DcR2, and osteoprotegerin (55, 56), and development of resistance (49). There are some ongoing clinical trials with TRAIL-based therapies such as the TRAIL-receptor agonists ABBV-621 in combination with bortezomib and dexamethasone (NCT04570631) and INBRX-109 alone (NCT04950075) or in combination with DNA damaging agents (NCT03715933) (49). No trials are currently investigating the combination of TRAIL-based therapy with DDRi agents.

Combination therapies may overcome some of these limitations, and various preclinical investigations support the combination of TRAIL-based therapies with DDRi. For example, one study found that PARPi enhanced the efficacy of a DR5 antibody in a pancreatic cancer mouse xenograft model (57). Others found that DNA-PKi potentiates p53-dependent apoptosis after treatment with a DNA damaging agent in AML cells, and that the TRAIL pathway plays a major role in this apoptotic response (58). Others have described upregulation of TRAIL-mediated apoptosis after ATMi treatment of melanoma cells (59). These findings provide rationale for combining TRAIL agonists and DDRi therapy in the clinic to enhance induction of apoptosis. Whether or not these types of combination treatments will enhance the anti-tumor immune response remains to be investigated.

## DDRi-induced upregulation of PD-L1

PD-L1 is a ligand that binds programmed cell death protein 1 (PD-1) on activated T cells. Binding of PD-L1 to PD-1 inhibits T cell activity (60) and elevated expression of PD-L1 is a major biomarker of favorable response to immune checkpoint inhibitors (61). Experimental evidence suggests that PARPi agents can upregulate PD-L1 expression by blocking glycogen synthase kinase-3 beta (GSK3β), a regulator of glycogen metabolism, cell cycle, inflammation, and proliferation (62). GSK3β also plays a role in the repair of both single- and double-stranded DNA breaks. PARPi-induced inhibition of GSK3β causes inhibition of DNA damage repair and upregulation of PD-L1 (63–65).

ATR inhibitors, on the other hand, seem to upregulate PD-L1 mRNA but downregulate PD-L1 protein expression (66–68). Further, studies have shown that DNA damage-induced upregulation of PD-L1 by cisplatin or ionizing radiation was suppressed by co-administration with ATRi agents (67). DNA-PK inhibitors seem to upregulate PD-L1 (69) along with WEE1 inhibitors (2) and Chk1/2 inhibitors (70, 71), likely by preventing the repair of double-stranded DNA breaks, which activates STAT1/3 signaling through ATM/ATR/Chk1 kinases, resulting in an upregulation of PD-L1 levels (60).

## PARP inhibitors

Poly-ADP ribose polymerase (PARP) is an enzyme that plays a critical role in the DNA repair pathways NER and BER, which repair DNA damage that is caused by therapeutic agents such as alkylating agents and chemotherapy (72). The PARP family contains 17 different proteins, but most studied are PARP1 and PARP2. PARP1 binds to DNA regardless of phosphorylation state and PARP2 preferentially binds phosphorylated DNA breaks, but otherwise these proteins largely function similarly (73). PARP inhibitors (PARPi) lead to death by synthetic lethality in cancer cells with deficiencies in the homologous recombination (HR) DNA repair pathway (74). PARPi is FDA-approved to treat breast cancer, prostate cancer, and gynecologic cancers including ovarian cancer (75–77).

PARP plays an important role in the normal functioning of the immune system. PARP2 contributes to the development of mature CD4+ and CD8+ T cells and *in vivo* data suggests that dual inhibition of PARP1 and PARP2 leads to a measurable decrease in T cell populations. PARP1 and PARP2 also contribute to normal T cell functioning as demonstrated by experiments in which PARP1/2 inhibition resulted in decreased IL-2 and IFN-γ-secreting T cells. PARP1 is also responsible for marking Foxp3-expressing T regulatory cells (T regs) for degradation. Additionally, PARP1 regulates NFAT, a family of transcription factors that that regulates CD4+ T cell differentiation, but it is unclear if inhibition of PARP1 biases CD4+ cells toward a Th1 or Th2 phenotype (73). Lastly, PARP1 may cooperate with IFI16 to induce noncanonical STING activation in response to chemotherapy-induced DNA damage (31).

Interestingly, in BRCA1-deficient ovarian cancer models, PARP inhibition with olaparib increased CD4+ and CD8+ T cells in the tumor and in circulation, reduced their expression of inhibitory receptors PD-1, Tim-3, and Lag-3, and increased their levels of TNF-alpha and IFN-γ secretion. In dendritic cells, PARPi upregulates costimulatory molecules CD80/CD86 and MHC class II which enhances antigen presentation and interactions with T cells. PARPi may increase expression of cell death receptor ligands and NKG2D ligands, which increases cancer cell sensitivity to NK cell-mediated killing. In macrophages, the impact of PARPi is dependent on factors in the tumor microenvironment including certain cytokines. The DNA damage caused by PARPi leads to cytosolic DNA, activating the cGAS/STING pathway and the type I IFN response (73). PARPi can also increase the amount of DNA in the cytosol, leading to the accumulation of neoantigens (78).

Due to the immune-stimulating properties of PARPi therapy, there is clinical interest in combining PARPi with immunotherapy. Clinical trials testing such combinations are ongoing for ovarian, ovarian, lung, urothelial, prostate, and gastrointestinal cancers (78). The results of these trials have been most promising in ovarian and breast cancer. In ovarian cancer, overall response rates (ORR) ranged from 45-63% and disease control rate (DCR) was 73-81% depending on the patient population. In breast cancer, ORR was 53% and DCR was 47-83% depending on patient population. PARPi alone is effective in patients with prostate cancer and has been combined with IT in several clinical trials. Results of the completed trials have been promising, with 9/17 patients with metastatic castration-resistant prostate cancer (mCRPC) treated with durvalumab and olaparib experiencing a PSA decline of >50% and 4/17 patients experiencing a radiographic response. A combination of pembrolizumab and olaparib in a cohort of patients with wild-type HR proteins had slightly less exciting results, with 7% partial response and 29% DCR. Studies in gastric cancer combining durvalumab and olaparib have reported a 10% ORR and 12-week DCR of 26% (78).

## DNA-PK inhibitors

DNA-PK is a serine/threonine protein kinase that plays a critical role in the DNA repair pathways classical NHEJ and HR. DNA-PK inhibitors (DNA-PKi) interfere with its kinase function and sensitize cells to DNA-damaging agents. DNA-PKi can be used as a single agent in some cancers with ATM deficiency by inducing synthetic lethality (79). No DNA-PKi therapies are FDA approved, however there are several ongoing clinical trials involving compounds such as XRD-0394, CC-115, VX-984 (M9831), AZD7648, and M3814 (nedisertib, peposertib, MSC-2490484A) to treat various type of cancer, typically advanced solid tumors (80, 81).

DNA-PK phosphorylates cGAS and suppresses its enzymatic activity. DNA-PK inhibition or deficiency correlates with decreased levels of phosphorylated cGAS and promotes antiviral immune responses (82). Additionally, as DNA-PK is critical to maintaining genomic stability, the loss or inhibition of this kinase may lead to high mutation load secondary to the development of genomic instability. Mutation of the gene encoding DNA-PK protein *PRKDC* is associated with high mutation load or microsatellite instable (MSI)-high status in The Cancer Genome Atlas pan-cancer cohort. Further, *PRKDC* knockout and DNA-PKi enhanced the efficacy of ICI (83, 84). The DNA-PKi AZD7648 sensitizes mice with colorectal tumors or melanoma to radiotherapy and induces a tumor control that is dependent on type I IFNs. There are phase I/II clinical trials involving AZD7648 in combination with chemotherapy (NCT03907969) and radiotherapy (NCT04550104) currently ongoing (85). Due to the dependence of AZD7648 on type I IFN responses, it would be interesting to combine this drug with immunomodulatory drugs that enhance the type I IFN response. Additionally, the DNA-PKi peposertib enhanced RT-induced TGFβ/PD-L1-targeted immunotherapy in mice, further supporting the combination of DNA-PKi, RT, and immunotherapy (69).

Three clinical trials are evaluating the combination of the DNA-PKi M3814 combined with the anti-PD-L1 ICI avelumab (86). M3814 has demonstrated monotherapy activity in several tumor cell lines, and M3814 + radiotherapy (RT) combined with avelumab significantly delayed tumor growth as compared to either agent alone + RT in MC38 syngrafts, indicating the benefit of combining DNA-PKi and immunotherapy (87). One trial is investigating M3814 and avelumab +/- radiotherapy for treatment of patients with advanced solid tumors (NCT03724890) (87). Another is investigating avelumab and RT +/- M3814 in advanced solid tumors and hepatobiliary malignancies (NCT04068194) (88). Lastly, one trial is evaluating RT vs. RT + M3814 vs. RT + M3814 + avelumab in patients with advanced prostate cancer that is unresponsive to hormonal therapy (NCT04071236) (89).

## WEE1 inhibitors

The WEE1 kinase family consists of three serine/threonine kinases: WEE1, PKMYT1, and WEE1B (WEE2). WEE1 and PKMYT1 play a crucial role in cell cycle regulation and DNA damage repair, while WEE2 regulates cell cycle progression and largely regulates meiosis. WEE1 and PKMYT1 can act like oncogenes and are a major focus in anti-cancer drug development (90). One WEE1 inhibitor, ZN-c3, has been granted fast track designation by the FDA for treatment of patients with uterine serous carcinoma. Another WEE1 inhibitor adavosertib (AZD1775, MK-1775) is highly developed and has been included in over fifty clinical trials to treat various types of cancer since 2008 (28).

WEE1 overexpression abrogates immune cell killing, for example by protecting cancer cells from granzyme B/TNFα induced cell death. One study found that cancer cells develop resistance to granzyme B/TNFα-mediated cytotoxic T cell killing by activating the G2/M cell cycle checkpoint. Further, they found that administration of WEE1i adavosertib reversed this effect, enhanced T cell killing, and synergized with an anti-PD-1 monoclonal antibody in murine models of oral cavity carcinoma, melanoma and colon adenocarcinoma with various TP53 mutations (91). WEE1 inhibition activates the STING and STAT1 pathways in SCLC and enhances the antitumor immune response to PD-L1 inhibition (2). Like the STING pathway, the STAT1 pathway is a major contributor to the anti-tumor immune response. Along with STAT2, STAT1 induces IFN-regulated genes, enhances antigen presentation, and contributes to an inflammatory, anti-cancer response. It is important to differentiate STAT1 and STAT2 from other STAT family members such as STAT3 and STAT5, which contribute to cancer cell survival, proliferation, and angiogenesis (3). It has also been shown that WEE1 induces anti-tumor immunity by activating endogenous retroviral elements and the dsRNA pathway (92). WEE1i also sensitizes head and neck cancers to natural killer (NK) cell therapies (93).

One ongoing clinical trial is evaluating adavosertib with the anti-PD-L1 ICI durvalumab for treatment of patients with advanced solid tumors (NCT02617277). DCR for the total cohort was 36%, suggesting antitumor activity (94). Notably, adavosertib + immunotherapy has a better safety profile compared to adavosertib + chemotherapy, warranting continued investigation (95). Another actively recruiting trial will test the safety, tolerability, efficacy, pharmacokinetics and pharmacodynamics of ZN-c3 alone and in combination with other drugs including the anti-PD-1 ICI pembrolizumab (NCT04158336) (27). As p53 mutations and overexpression of SKP2 and CUL1 may be biomarkers of a favorable response to WEE1i, additional clinical trials in these patient populations in combination with immunotherapy are needed (27, 91).

## CHK1/2 and ATR inhibitors

ATR and its major downstream effector checkpoint kinase 1 CHK1 play a role in the DNA damage response. In response to single-stranded DNA breaks, ATR activates CHK1 to trigger intra-S and G2/M phase checkpoints. In response to double-stranded DNA breaks, the MRE11/NBS1/RAD5 complex activates ATM and CHK2 to trigger the G1/S-phase checkpoint (96). Because ATR has a broader range of biological functions than CHK1, it is thought that ATRi may have greater toxicity in normal cells. Therefore, the clinical development of CHK1i is more advanced than ATRi (96). There are over twenty CHK1/2 and ten ATR inhibitors in various stages of clinical trials for many different cancer types mostly in combination with chemotherapy but also with RT and histone deacetylase inhibitors (HDACi) (96). No CHK1/2 or ATR inhibitors are FDA-approved yet (97).

One study found that in the leukemia cell line THP-1, CHK1i increased TBK1 but did not increase IRF3 phosphorylation, induce IRF3 or NF-κB reporter activation, nor induce a type 1 IFN response (98). The same group found that in solid tumor cell lines, addition of CHK1i to chemotherapy treatment such as gemcitabine or camptothecin increased the accumulation of cytosolic DNA but decreased the level of chemotherapy-mediated IRF1 and STAT1 phosphorylation. Interestingly, similar results as far as lack of type 1 IFN response were found using ATRi and WEE1i, indicating that context such as cancer type may affect the ability of DDRi to induce the cGAS/STING pathway (99). Another study found that in murine melanoma models, CHK1i induces an immunogenic signaling and increased levels and activity of CD8+ T cells (100). Similarly, others observed that treatment of patients with head and neck squamous cell carcinoma with CHK1i led to an upregulation of transcripts associated with T-cell activation and inflammatory cytokines and chemokines but also T regs (101). Interestingly, others have shown that the combination of CHK1i and ionizing RT increases micronuclei formation and induces an abscopal tumor regression response in a murine melanoma model (102).

Despite the advanced preclinical development of CHK1i alone or in combination with chemotherapy or RT, there are currently no ongoing or completed clinical trials testing the combination of CHK1/2i with immunotherapy. There are three trials that are evaluating ATRi with immunotherapy. One study (NCT04576091) is investigating sensitization to pembrolizumab with the ATRi elimusertib in combination with RT for treatment of patients with recurrent head and neck cancer. As of February 2022, no patients were enrolled in this study. Another trial is currently recruiting patients with advanced solid tumors to evaluate elimusertib + pembrolizumab without RT (NCT04095273). Lastly, one trial will evaluate the combination of ATRi M1774 with immune checkpoint inhibition for treatment of patients with metastatic or locally advanced unresectable solid tumors (NCT05396833). The results of these trials are highly anticipated.

## ATM inhibitors

ATM is activated by double stranded breaks in DNA, and cells that are deficient in ATM experience abnormal DNA repair. Activated ATM phosphorylates p53 at serine 15 to activate it and phosphorylates MDM2 to prevent its inhibitory binding to p53. ATM also phosphorylates and activates CHK2, which phosphorylates p53 at another activating site (serine 20). p53 induces p21 to inhibit CDK2/cyclin E to induce arrest at the G1 phase of the cell cycle. Activated ATM also phosphorylates NBS1, which is necessary for RT-induced S phase cell cycle arrest, but the complete mechanism remains to be clarified (103).

In *Drosophila* models, ATR deficiency causes an innate immune response (104). In murine and human cancer cell lines, ATM deficiency induces ISG expression and tumor infiltration of immune cells in a cGAS/STING-dependent manner. Further investigation revealed this effect was mediated specifically by leakage of mitochondrial DNA rather than nuclear DNA into the cytoplasm. The same group found that ATM expression levels negatively correlate with type 1 IFN gene expression in human tumor tissues and that patients with tumors harboring ATM mutations have a favorable response to ICI (105). Similar findings as far as ATM mutations serving as a biomarker of favorable response to ICI have been made in bladder cancer (106). Other studies in pancreatic cancer have shown that ATMi induces type 1 IFNs in a cGAS/STING-independent manner, but this response was dependent on TBK1 and SRC (107). Despite this preclinical evidence of ATMi-induced immune stimulation, there are no clinical trials testing the combination of ATMi and immunotherapy.

## Inhibition of oxidative damage repair

Chemotherapy and radiation therapy are well-known inducers of oxidative stress, a condition in the cell characterized by excess reactive oxygen species and the resulting processes that detoxify the cell and repair oxidative damage (108). Oxidative stress plays a major role in inducing cellular damage after treatment with DNA-damaging agents (109). Oxidative stress increases levels of intracellular Ca^2+^, induces Fenton reaction DNA lesions, and triggers DNA repair mechanisms (110). BER plays a major role in the cellular response to oxidative DNA damage. During BER, damaged bases are excised, generating apurinic/apyrimidinic (AP) sites. At these sites, apurinic/apyrimidinic endonuclease 2 (APE2, APN2, or APEX2) creates a single-strand break which is then fixed by other DNA repair enzymes. Thus, APE2 plays a critical role in the repair of oxidative damage, and in fact knockdown of APE2 led to increased micronuclei formation in the PANC1 pancreatic cancer cell line (111). Oxidative stress plays many roles in the immune microenvironment of the tumor (112). For example, APE2 is involved in B cell development and immunoglobulin class switch recombination and APE2-knockout mice develop defects in immune responses. BER and ATR pathways, both of which are heavily involved in regulating PD-L1 expression, rely on APE2. APE2 involvement in the response to immunotherapy is likely but has not been investigated. There are no APE2 inhibitors in clinical trials for the treatment of cancer. Future studies should investigate the impact of APE2 and other oxidative damage repair enzymes on the immune response and response to immunotherapy (113).

## Conclusion and open questions

The clinical applicability of DDRi has been clearly demonstrated for cancers with deficiencies in the DDR pathway. The combination of DDRi with DNA-damaging agents has improved the efficacy of these agents in certain contexts. It is now well-recognized that DDRi compounds stimulate the immune system against cancer and that this effect may be enhanced by combinations with DNA-damaging agents. The cGAS/STING pathway is a major regulator of DDRi-induced immune stimulation, though the STAT1 pathway, TRAIL pathway, and direct activation of anti-cancer immune cells also play important roles. The effects of DDRi on the immune system provide rationale for their combination with immunotherapy such as ICI, as is being tested in various clinical trials.

TRAIL is induced by the IFN-γ/STAT1 and cGAS/STING pathways (7, 40–42) that are activated after DDRi therapy. TRAIL-based therapies have therapeutic potential because of their ability to induce both apoptosis and lasting anti-cancer immunity. There are no FDA-approved TRAIL-based treatments (49), however numerous clinical trials are continuing to investigate new approaches and combination treatments (49, 55, 56). Many preclinical investigations support the combination of TRAIL-based therapies with DDRi (57–59), providing rationale for clinical translation. The addition of ICI to this treatment regimen should also be considered given the heavy involvement of both DDRi and the TRAIL pathway in anti-cancer immune activation.

Less than half (12/33) of the clinical trials that are testing combinations of DDRi and immunotherapy involve combination with a DNA damaging agent such as chemotherapy or radiotherapy (**Table 1**). Most of the trials that do not include a DNA damaging agent are for treatment of malignancies in which alterations in DDR proteins are common, such as breast cancer and ovarian cancer. While DDRi therapy can induce accumulation of cytosolic DNA and stimulate the immune system in the presence of these alterations, co-administration of DNA damaging agents should be considered to expand the use of this combination therapy to patients without such alterations.

Cancer stem cells are cancer cells with stem-like phenotypes that are slow-cycling and have highly efficient DNA repair, which grants them resistance to chemotherapy and radiotherapy (114). Cancer stem cells present a major challenge as far as overcoming drug resistance and cancer recurrence. Cancer stem cells may also be able to evade the immune system (115), thus immunotherapy alone may not be active against this subset of the tumor. Inhibiting the highly efficient DNA repair processes in cancer stem cells, especially in combination with DNA-damaging agents, may be a promising approach to eliminate this cell population (116). Bulk tumor reduction and elimination of cancer stem cells with the combination of DDRi and DNA damaging agents sequenced with immunotherapy for lasting tumor regression may be a viable treatment option for patients with tumors characterized by cancer stemness. Optimization and validation of treatment dose, timing, and sequencing is necessary *in vivo*.

Another area in need of further investigation is the differential effects of various DDRi agents on the immune system. Though inhibition of each DDR protein has similar effects in most studies, there seem to be context-specific differences especially for CHK1i. Similar context-dependency may be found with complementary study of other DDR proteins. Further investigation is critical to the application of DDRi + immunotherapy to wider patient populations.

## Author contributions

Conceptualization, WE-D and LC. Investigation, WE-D and LC. Writing—original draft preparation, LC. Writing—review and editing, WE-D and LC. Supervision, WE-D. Project administration, WE-D. Funding acquisition, WE-D. All authors have read and agreed to the published version of the manuscript.

## Funding

This work was funded by the Teymour Alireza P’98, P’00 Family Cancer Research Fund, grant number GFT640730, established by the Alireza Family.

## Conflict of interest

The authors declare that the research was conducted in the absence of any commercial or financial relationships that could be construed as a potential conflict of interest.

## Publisher’s note

All claims expressed in this article are solely those of the authors and do not necessarily represent those of their affiliated organizations, or those of the publisher, the editors and the reviewers. Any product that may be evaluated in this article, or claim that may be made by its manufacturer, is not guaranteed or endorsed by the publisher.

## Abbreviations

ATM, ataxia telangiectasia mutated; ATR, ataxia telangiectasia and Rad3 related; BER, base excision repair; cGAS, cyclic GMP-AMP synthase; CHK1/2, checkpoint kinase 1/2; DCR, disease control rate; DDR, DNA damage response; DNA-PK, DNA-dependent protein kinase; DR5, death receptor 5; HR, homologous recombination; ICI, immune checkpoint inhibition; IFN, interferon; ISG, interferon stimulated gene; MDSC, myeloid-derived suppressor cell; MSI, microsatellite instable; NER, nucleotide excision repair; NHEJ, non-homologous end joining; NK, natural killer; ORR, overall response rate; PARP, poly-ADP ribose polymerase; PD-1, programmed cell death-1; PD-L1, programmed death-ligand 1; RT, radiation therapy; SASP, senescence-associated secretory phenotype; STAT1, signal transducer and activator of transcription 1; STING, stimulator of interferon genes; TNF, tumor necrosis factor; TRAIL, TNF-related apoptosis-inducing ligand.
